# Biological clock regulation by the *PER* gene family: a new perspective on tumor development

**DOI:** 10.3389/fcell.2024.1332506

**Published:** 2024-05-15

**Authors:** Kai Chen, Yaohui Wang, Dengxiong Li, Ruicheng Wu, Jie Wang, Wuran Wei, Wei Zhu, Wenhua Xie, Dechao Feng, Yi He

**Affiliations:** ^1^ Department of Urology, The First Hospital of Jiaxing, The Affiliated Hospital of Jiaxing University, Jia Xing, China; ^2^ Department of Urology, Institute of Urology, West China Hospital, Sichuan University, Chengdu, China; ^3^ Department of Urology, The Third Medical Center of PLA General Hospital, Beijing, China; ^4^ Division of Surgery and Interventional Science, University College London, London, United Kingdom

**Keywords:** period gene, circadian rhythm, carcinogenic effect, biological behavior, cancer therapy

## Abstract

The Period (*PER*) gene family is one of the core components of the circadian clock, with substantial correlations between the *PER* genes and cancers identified in extensive researches. Abnormal mutations in *PER* genes can influence cell function, metabolic activity, immunity, and therapy responses, thereby promoting the initiation and development of cancers. This ultimately results in unequal cancers progression and prognosis in patients. This leads to variable cancer progression and prognosis among patients. In-depth studies on the interactions between the *PER* genes and cancers can reveal novel strategies for cancer detection and treatment. In this review, we aim to provide a comprehensive overview of the latest research on the role of the *PER* gene family in cancer.

## 1 Introduction

With population aging, it is projected that by 2030, almost 20% of the world’s population will be over 65, and by 2050, this figure is expected to reach 1.6 billion (Feng et al., 2023a). Cancers, closely associated with aging, are characterized by aberrant cellular proliferation and differentiation, continuing to pose a significant threat to global health (Hanahan, 2022; Shen et al., 2022; Feng et al., 2023b; Wang et al., 2023). In 2020, approximately 19 million new cancer cases and over 10 million cancer-related deaths were recorded worldwide (Sung et al., 2021). Specifically, in China, there were 4.57 million new cancer cases and 3.00 million cancer-specific deaths in the same year (He and Ke, 2023). Traditional cancer treatments include radiation therapy, chemotherapy, and surgery (Jin et al., 2022; Sirhan et al., 2022; Xing et al., 2022; Association, 2023), while molecularly targeted therapies and immune checkpoint inhibitors have transformed oncology (Chen et al., 2022; Chan et al., 2023; Yin et al., 2023; Yu et al., 2023). Despite advancements, the adverse effects of treatments on patient survival and quality of life remain pressing challenges in cancer therapy (Zhang and Zhang, 2020; Peng et al., 2022), complicating patient management (Wang YH. et al., 2020; Mokhtari-Hessari and Montazeri, 2020). High-throughput sequencing, a pioneering molecular biology technique, has fueled new oncology research directions (Walter et al., 2022; Larson et al., 2023). Contrasting with the conventional American Joint Committee on Cancer staging, which relies on tumor size, lymph node status, and metastasis, new classifications based on tumor genetic expression patterns have emerged, correlating more closely with clinical outcomes and patient survival (Yin et al., 2020; Park et al., 2022; de Jong et al., 2023). This shift signifies a move towards personalized precision medicine in oncology, with the potential to uncover new therapeutic targets and prognostic biomarkers through a deeper understanding of molecular mechanisms in cancer (Hong et al., 2020; Bao et al., 2023).

The Earth’s rotation generates environmental variables with a 24-h periodicity, including temperature and light fluctuations (Hou et al., 2022; Laosuntisuk et al., 2023). Various life forms, including animals, plants, bacteria, fungi, have developed intricate internal timing mechanisms to synchronize their behavior and physiological processes with these cyclic environmental changes (Patton and Hastings, 2023). These mechanisms are known as “circadian rhythms,” a term derived from the Latin “circa diem”, meaning “about a day” (Allada and Bass, 2021). Thus, circadian rhythm denotes an organism’s inherent rhythm-regulating system. In humans and other mammals, this 24-h rhythm is orchestrated by the circadian clock (Huang et al., 2023). The suprachiasmatic nucleus (SCN) of the hypothalamus, acting as the central clock, utilizes neuroendocrine pathways to coordinate peripheral clocks throughout the body (Starnes and Jones, 2023; Zhang-Sun et al., 2023). This network of clocks ensures the consistency of vital functions and numerous physiological activities.

## 2 Molecular circadian clock and cancer connection

**FIGURE 1:**
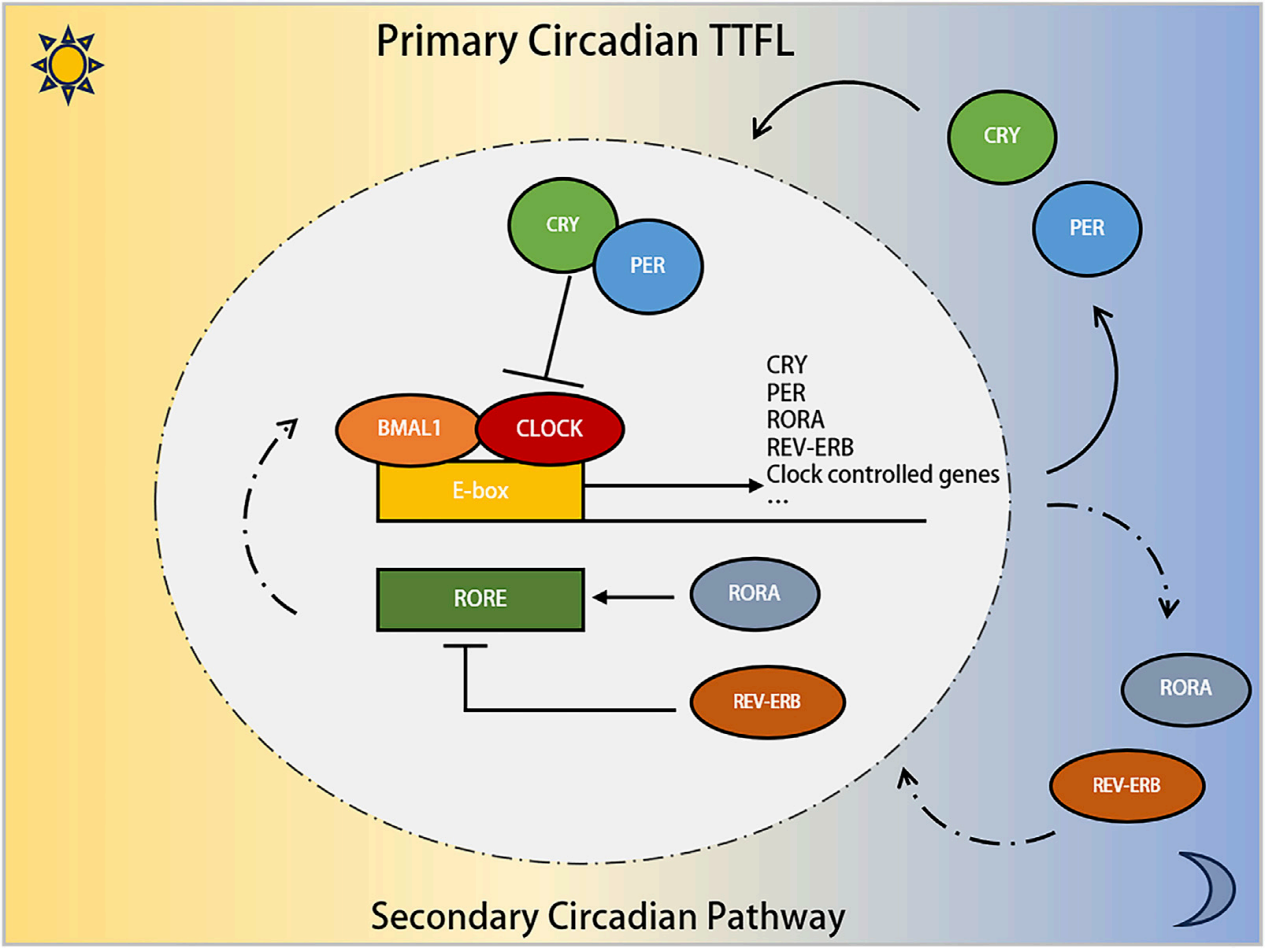
The involvement of the *PER* gene family in the molecular mechanism of Circadian Clock. The core circadian transcriptional machinery consists of the positive transcription factors (CLOCK and BMAL1 proteins), and the repressors factors (PER and CRY proteins). Additionally, the nuclear receptors RORα, and REV-ERB compose the secondary circadian pathway.

From a molecular perspective, the circadian clock in mammals is governed by a transcription-translation feedback loop (TTFL), involving circadian genes and associated proteins (Patke et al., 2020; Li et al., 2023). This process begins when Brain and Muscle ARNT-Like Protein 1 (BMAL1) and Circadian Locomotor Output Cycles Kaput (CLOCK) proteins form a heterodimer that binds to the E-box in the promoter regions of various genes (Cox and Takahashi, 2019). This includes clock-controlled genes (CCGs) and inhibitory elements like Period (PER) and Cryptochrome (CRY) proteins (Cao et al., 2021). As PER and CRY proteins levels rise, they eventually inhibit CLOCK-BMAL1 activity, thus regulating their own synthesis and setting the stage for a new circadian cycle. Additionally, RORα and REV-ERBs modulate BMAL1 expression through their interaction with REV-ERB-ROR response elements, creating a secondary loop (Preitner et al., 2002; Sato et al., 2004). D-box binding protein (DBP) and E4 promoter-binding protein 4 (E4BP4/NFIL3) also modulate gene expression and CCG activity via D-box promoters (Ripperger and Schibler, 2006). Furthermore, post-translational modifications (PTMs) by kinases/phosphatases and the ubiquitin-proteasome system, including Casein kinase Ⅰ epsilon (CKⅠε), β-transducin repeat-containing protein (β-TrCP), F-box and leucine-rich repeat protein 3 (FBXL3), and Tumor necrosis factor receptor-associated factor-2 (TRAF2) (Eide et al., 2005; Reischl et al., 2007; Siepka et al., 2007; Chen et al., 2018), maintain the stability and function of clock proteins. These mechanisms collectively create a delayed negative feedback loop, producing the roughly 24-h circadian rhythm. Our team has previously detailed this molecular mechanism in extensive studies (Feng et al., 2022; Zhu et al., 2023) (Figure 1).

Given the pivotal role of circadian rhythm in regulating diverse biological functions, extensive research has shown that its disruption is associated with a heightened risk of various diseases, including cardiovascular disorders, neurological conditions, and cancers (Crnko et al., 2019; Sancar and Van Gelder, 2021; Niu et al., 2022). In 2007, the World Health Organization identified shift work that disrupts circadian rhythms as a Group 2A carcinogen (Straif et al., 2007). Epidemiological studies have indicated that long-term night shift workers face increased risks of certain cancers, such as breast, prostate, and lung cancer (Cordina-Duverger et al., 2022; Berge et al., 2023; Schernhammer et al., 2023). Remarkably, about 43% of protein-coding genes in the mammalian genome are regulated by circadian clock genes, which are integral to various critical physiological and pathological processes (Zhang et al., 2014). Alterations in circadian clock genes or their functions can lead to disrupted cellular activities linked with cancer hallmarks, including cell cycle disruption (Lévi et al., 2007; Qu et al., 2023), genomic instability (Zhang et al., 2023), metabolic reprogramming (Chun et al., 2022), and immune system dysregulation (Chen et al., 2020; Zhang et al., 2024). As a result, the link between circadian clock genes and cancer has become a focal point in oncological research. In this review, we concentrate on the *PER* gene family, a central component of the circadian clock, explore its connection to cancer and summarize current research findings in the area.

## 3 Brief overview of the *PER* gene family

**FIGURE 2:**
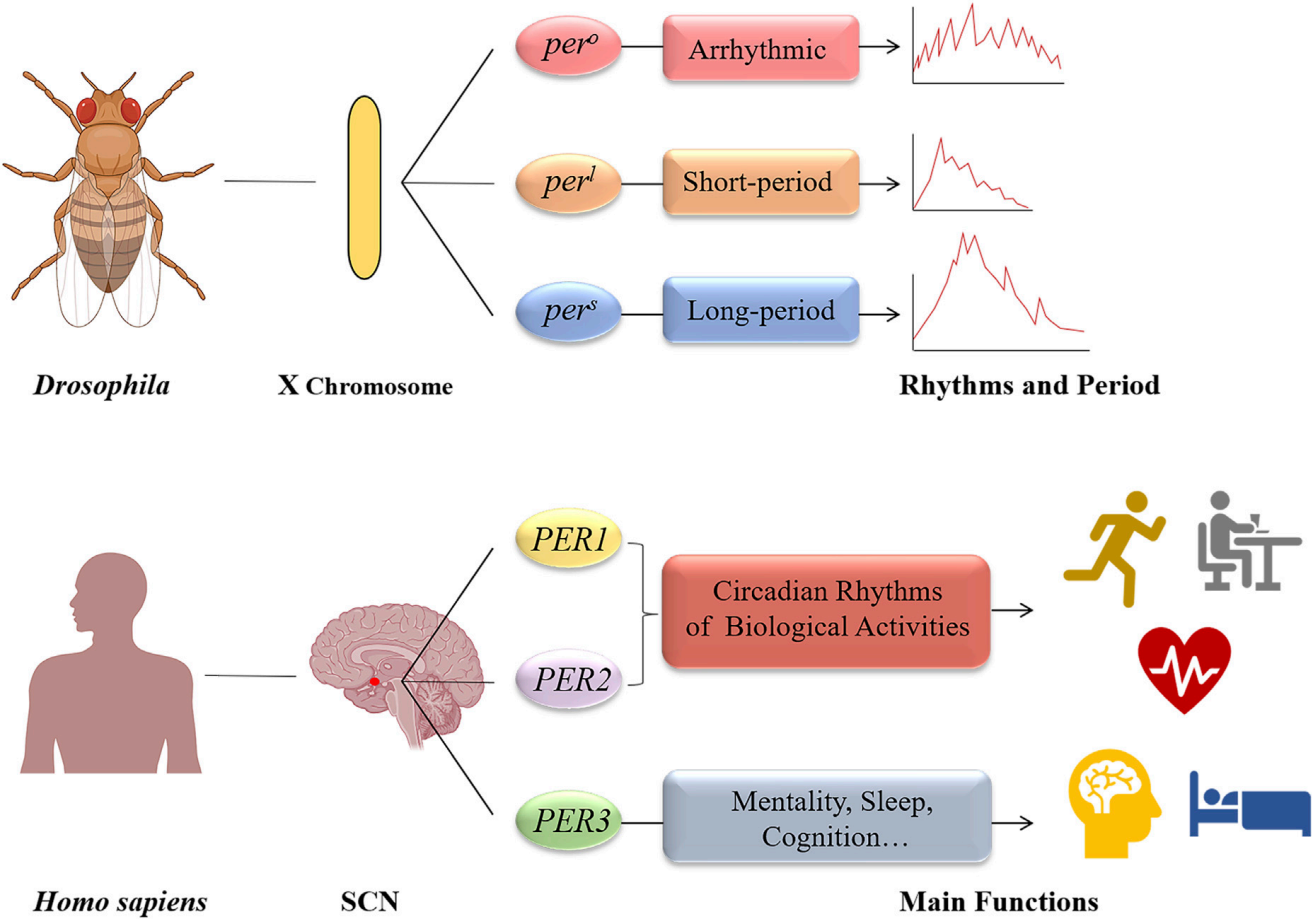
The period genes of *Drosophila melanogaster* and human. The discovery of the period gene can be traced back to 1971, when Konopka and Benzer identified three gene mutations (*per* ^*o*^, *per* ^*s*^, and *per* ^*l*^) on the X chromosome of *Drosophila melanogaster*; As for human, there are three homologous genes (*PER1*, *PER2*, and *PER3*). The *PER1* and *PER2* genes are primarily responsible for maintaining circadian rhythms of basic biological activities. And *PER3* gene play an important role in mentality, sleep, and cognition.

There are three homologous genes in mammals (*PER1*, *PER2*, and *PER3*) that are integral to the *PER* gene family, a key component of circadian clock genes (Deng and Yang, 2019). When Konopka and Benzer induced mutations in *Drosophila melanogaster* using ethyl methane sulfonate in 1971, they observed three distinct rhythm patterns in the mutants’ eclosion and locomotor activities. The *per* gene was subsequently located on the X chromosome of *D. melanogaster*, with its variants (*per* ^*o*^, *per* ^*s*^, and *per* ^*l*^) associated with these rhythm changes (Konopka and Benzer, 1971). In 1984, the *per* gene was isolated and cloned by the teams of Jeffrey C. Hall (Zehring et al., 1984), Michael Rosbash (Reddy et al., 1984), and Michael Young (Bargiello and Young, 1984), who later received the 2017 Nobel Prize in Physiology or Medicine for their pioneering work on circadian rhythms. Further research by Hall and Rosbash demonstrated that the Per protein was a nuclear protein that oscillated between the cytoplasm and the nucleus, influencing the expression of its mRNA, leading to the hypothesis that the *per* gene acts as a transcription factor with feedback regulation (Zeng et al., 1994; Yildirim et al., 2022). Additionally, Young and his team found that the *per* gene’s oscillation was synchronized with another clock gene, *Timeless* (*tim*), and that mutations in *tim* significantly affected the Per protein’s synthesis, phosphorylation, and transport (Sehgal et al., 1995; Ahmad et al., 2021). These findings elucidated the interaction between Tim and Per proteins, supporting the TTFL model. Subsequent discoveries showed that CLOCK and BMAL1 proteins form a dimer that binds to the E-box to transcriptionally regulate the *PER* genes, confirming their role as positive regulators in the circadian rhythm (Vitaterna et al., 1994). The identification of other core clock genes, such as *CLOCK* (Allada et al., 1998), *CYC* (Rutila et al., 1998), and *CRY* (Todo et al., 1996), further elaborated the TTFL model, enhancing our understanding of circadian gene transcription regulation. These advancements propelled researches into circadian rhythms, underscoring the significance of clock genes in human physiology and diseases (Vitaterna et al., 2019) (Figure 2).

The *PER* gene family plays a pivotal role in maintaining circadian rhythms. Studies have demonstrated that *Per2* homozygous mutant mice exhibit significantly shorter circadian rhythms than wild-type mice, which disappear in constant darkness (Zheng et al., 1999). Likewise, mice with homozygous *Per1* mutations also display shorter rhythms, with *Per2* influencing clock gene expression rhythm through transcriptional regulation (Zheng et al., 2001). Shiromani and colleagues found that *Per*1/*Per*2 double mutant mice quickly experienced a decrease in core body temperature rhythm and a reduced circadian period, while *Per3* mutants showed no notable rhythm alterations (Shiromani et al., 2004). Nakamura et al. (Nakamura et al., 2023) observed that the triple knockout of *Per1*/*Per2*/*Per3* severely disrupted the estrous cycle in C57BL/6J mice (melatonin deficient), potentially due to compromised SCN amplitude stability. While the contribution of *PER3* to circadian rhythm maintenance is less pronounced than *PER1* and *PER2*, it has been linked to nocturnal preferences, psychiatric disorders, sleep patterns, and cognitive functions (Chen et al., 2023). The *PER3* gene exhibits polymorphism in humans, with a variable number of tandem repeats (VNTR) consisting of a 54-base pair sequence in the 18th exon (Barragan et al., 2022). Aytac et al. (Aytac et al., 2022) identified an association between the VNTR variant at the *rs57875989* locus of the *PER3* gene and bipolar affective disorder risk, combining clinical scale assessments and blood analysis. The 4R/4R genotype appears to be protective against bipolar disorder, whereas the 5R/5R genotype is linked to a higher incidence of moderate manic symptoms in a study of 121 patients and controls (Yegin et al., 2021). Additionally, a significant correlation was found between the rs228697 SNP in *PER3* and increased vulnerability to sleep-wake disturbances in Alzheimer’s patients (Lozano-Tovar et al., 2023). Azevedo et al. (Azevedo et al., 2021) also noted a potential association between severe obesity and the rs228729 locus of the *PER3* gene.

## 4 The *PER* gene family aberration and cancers

Emerging evidence suggests that alterations in clock genes, including the *PER* gene family (*PER1*, *PER2*, and *PER3*), play a significant role in cancer development and progression due to their regulatory functions in biological cycles and physiological processes. Researches have increasingly focused on comparing the *PER* genes expression levels in tumor and normal tissues to elucidate the potential link between *PER* genes aberrations and cancers. Downregulations of *PER1*, *PER2*, or *PER3* have been observed in various cancers, including oral squamous cell carcinoma (Gong et al., 2021), head and neck squamous cell carcinoma (Li et al., 2019; Rahman et al., 2019), colorectal cancer (Orhan et al., 2019; Sahar et al., 2022), breast cancer (Liu et al., 2021; Liu et al., 2022a), ovarian cancer (Angelousi et al., 2019; Chen et al., 2021), melanoma (Lesicka et al., 2023), and hematological malignancies (Jiang et al., 2021). Studies have also highlighted the association between VNTR or SNPs polymorphism in the *PER* gene family and an increased risk of certain cancers, such as breast cancer (Fores-Martos et al., 2021; Song et al., 2023), colorectal cancer (Holipah and Kuroda, 2020), and prostate cancer (Wendeu-Foyet et al., 2019; Hinoura et al., 2021), through the analysis of tumor tissues or patient blood samples. To enhance our understanding of the prognostic significance of the *PER* gene family alterations in tumor progression, a growing body of research is exploring the relationship between these genetic changes and clinicopathological features.

A meta-analysis involving 7,476 cancer patients revealed that decreased *PER1* gene expression was associated with poorer tumor differentiation and greater invasion depth, whereas reduced *PER2* expression was correlated with advanced pathological stages and increased metastasis; furthermore, lower levels of both *PER1* and *PER2* were linked to shorter overall survivals (Zhang et al., 2020). Patients with advanced-stage head and neck squamous cell carcinoma exhibited markedly lower levels of PER1, PER2, and PER3 proteins compared to those with early-stage tumors, and higher levels of these proteins were associated with longer overall and recurrence-free survival. In oral squamous cell carcinoma, low *PER2* expression was connected to poor prognosis, tumor grade progression, and lymph node metastasis (Xiong et al., 2018), whereas in lung cancer, increased *PER2* expression was associated with less malignant differentiation and fewer lymph node metastases (Xiang et al., 2018). Transcriptome analysis from the GEO database indicated that *PER3* gene expression could predict outcomes for ER+/HER2-breast cancer patients through multifactor Cox analysis (Cadenas et al., 2014). The link between VNTR or SNP variations in the *PER* gene family and cancer risk has also attracted significant research interest (Morales-Santana et al., 2019). Lesicka et al. (Lesicka et al., 2019) identified that the dominant phenotype of *PER1* rs2735611 and the recessive phenotype of *PER2* rs934945 were associated with increased breast cancer risk. Additionally, the higher prevalence of the *PER2* VNTR 4R/3R and 3R/3R genotypes in pancreatic cancer patients compared to healthy controls suggested that a greater proportion of the 3R allele in the *PER2* VNTR may be a risk factor for pancreatic cancer (Dagmura et al., 2021).

**FIGURE 3:**
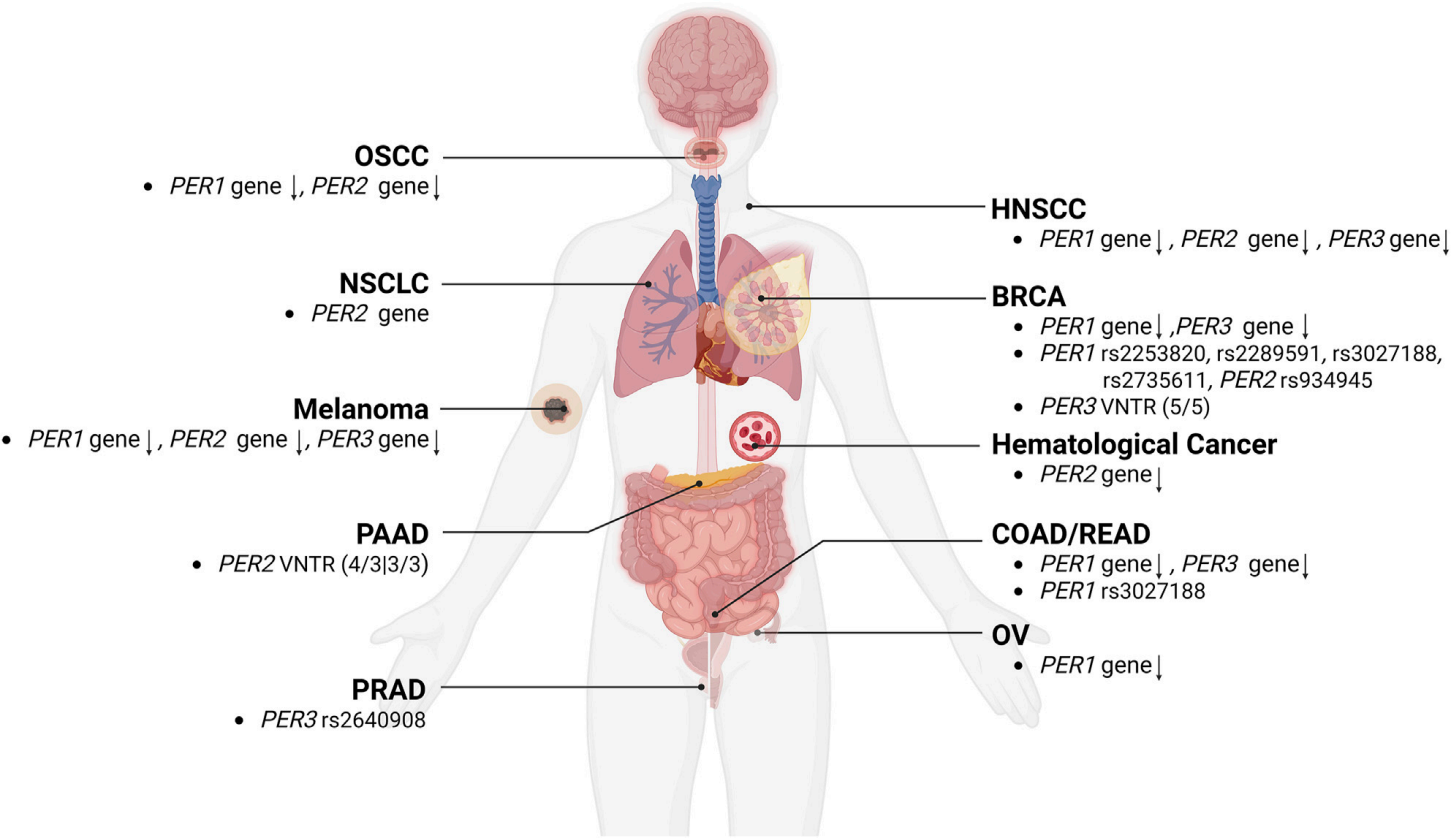
The relationships between alterations of the *PER* gene family and cancers. The aberrations, and VNTR or SNPs polymorphism of *PER1*, *PER2*, and *PER3* gene were correlated with the development of various types of cancers, including BRCA (breast cancer), COAD/READ (colorectal cancer), HNSCC (head and neck squamous cell carcinoma), melanoma, NSCLC (non-small cell lung cancer), OSCC (oral squamous cell carcinoma), OV (ovarian cancer), PAAD (pancreatic adenocarcinoma), and PRAD (prostate cancer).

Overall, the majority of previous cancer studies have consistently demonstrated significant downregulation of the *PER* gene family, often linked to reduced patient survival, poorer prognosis, and clinicopathological factors such as low tumor differentiation, advanced tumor stage, lymph node metastasis, and more aggressive tumor characteristics. Furthermore, researches into VNTR or SNPs polymorphisms of the *PER* gene family have established a connection between their anomalies and an increased risk of certain cancers (Figure 3).

## 5 The *PER* gene family and cell function

The cell cycle, which promotes cell division in a specific sequence and phases, exhibits a biological rhythm akin to the circadian clock. Cell cycle progression is governed by transient interactions between cyclins and cyclin-dependent kinases (CDKs), triggering phase transitions; While activities of the cyclin-CDK complexes are strictly regulated by CDK inhibitors like p16, p21, and WEE1, to halt the cell cycle under stress or damage conditions (Suski et al., 2021; Matthews et al., 2022). Consequently, there is growing interest in exploring the molecular interplay between the cell cycle and circadian rhythms, particularly regarding abnormal cell proliferation in cancers (Farshadi et al., 2020; Yao et al., 2021). For example, the PER1 protein has been shown to inhibit the cell cycle by interacting with checkpoint proteins ATM and CHK2 in colorectal cancer cells (Gery et al., 2006); similarly, NONO was found to influence the *p16-Ink4A* site by binding to PER1/PER2 proteins during the G1 phase, linking the cell cycle with the circadian rhythm (Kowalska et al., 2013). Conversely, the *TP53* gene can inhibit *PER2* gene activation by interfering with BMAL1/CLOCK-mediated E-box transcription, serving as a key cell cycle regulator (Miki et al., 2013; Zou et al., 2020; Engeland, 2022). Gao et al. (Gao et al., 2021) observed that *IDH1* gene mutations in gliomas significantly reduced *PER* genes expression (*PER1*, *PER2,* and *PER3*) and altered the expression of cell cycle-related proteins like Cyclin A, CDK2, and Cyclin D3, resulting in increase of G1 phase cells and decrease of S phase cells. Overexpressing the *PER2* gene in the chronic myelogenous leukemia cell line KCL22 led to G1 phase cell cycle arrest, while its downregulation expedited the transition to the S phase (Wang et al., 2020b; Basti et al., 2022). Recently, Han and colleagues integrated cell microarray analysis with experimental validation, revealing that overexpressing the *PER1* gene affected the expression of cell cycle-related proteins such as Wee1, CRE-BP1, CDK1, and GADD45A, thereby inhibiting the cell cycle in cholangiocarcinoma cells (Han et al., 2016).

The epithelial-mesenchymal transition (EMT), marked by increased vimentin and decreased E-cadherin expression, is a critical process where cells transition from epithelial to mesenchymal traits (Bakir et al., 2020; Liu et al., 2022b; Huang et al., 2022; Lee et al., 2022). EMT is essential for tumor initiation, enhanced migration, metastasis, and treatment resistance (Pastushenko and Blanpain, 2019). Studies have shown that EMT in glioma C6 and breast cancer MCF-7 cells correlated with enhanced circadian rhythms and increased *PER2* gene expression (De et al., 2020). Guo et al. (Guo et al., 2020) observed that elevated *PER2* gene protected oral cancer cells from EMT via upregulation of TP53 protein. Conversely, *PER2* knockdown in colorectal cancer cells activated the Snail/Slug-related EMT pathway, promoting proliferation and invasiveness of cancer cells (Xiong et al., 2022). Additionally, Lin et al. (Lin et al., 2020) reported that the mangiferin-induced EMT suppression in lung cancer cells was associated with decreased E-cadherin and increased PER1 protein expression. These findings suggest that the *PER1* and *PER2* genes play a role in inhibiting EMT, potentially reducing migration and invasion in cancer cells.

## 6 The *PER* gene family and metabolism

Metabolic reprogramming, a hallmark of cancer cells (Pavlova et al., 2022), involves altering cellular metabolism to manage different inputs and stressors (Wang et al., 2024). Cancer cells adapt their metabolic pathways to oncogenic mutations and external nutritional conditions, supporting their increased biosynthetic and energy needs while mitigating oxidative stress associated with survival and proliferation (Altea-Manzano et al., 2020; McGuirk et al., 2020; Schiliro and Firestein, 2021). An example is the Warburg effect, where cancer cells prefer glycolysis for energy production under aerobic conditions, highlighting their metabolic flexibility (Warburg et al., 1923; Warburg et al., 1924). Circadian rhythms regulate key biological processes associated with material and energy metabolism, including sleep-wake cycles (Daan et al., 1984; Wendrich et al., 2023), thermogenesis (Hasan et al., 2021), food intake (Teixeira et al., 2022), and glucose and lipid metabolism (Kalsbeek et al., 2010; Frazier et al., 2023; Small et al., 2023). Previous studies showed that mice lacking *Bmal1* or *Clock* genes exhibit significant defects in gluconeogenesis, lipid, and glucose metabolism (Bolshette et al., 2023). In *Drosophila*, dietary restriction prolonged lifespan via promoting fat metabolism, which linked to increased oscillations and expressions of Per and Tim proteins (Katewa et al., 2016). Research on transgenic mice with the h*PER2* S662G mutation revealed that derived lung cancer cells increased glucose, glutamine, and lactic acid consumption. Isotope tracing has also illustrated enhanced glucose utilization in cancer cell glycolysis and the tricarboxylic acid cycle (Papagiannakopoulos et al., 2016). Furthermore, Gong et al. (Gong et al., 2021) found that *PER1* gene knockdown raised levels of key glycolytic enzymes, boosting glucose uptake and lactate production in oral squamous cell carcinoma cells. These studies indicate that disrupting *PER1* or *PER2* gene functions in cancer cells can lead to elevated metabolism and energy production, supporting their rapid proliferation and adaptability.

## 7 The *PER* gene family and tumor immune microenvironment

The tumor microenvironment (TME) comprises various cellular components (such as immune cells, fibroblasts, and endothelial cells) and acellular elements (including cytokines, growth factors, and the extracellular matrix) (Ribeiro Franco et al., 2020). Innate and adaptive immune cells within the TME interact with cancer cells directly or through chemokine and cytokine signaling (Liu et al., 2023a; Xiong et al., 2023; Zetrini et al., 2023). These interactions significantly influence the biological behavior and therapeutic responses of cancer cells, impacting patients’ clinical outcomes and prognosis (Neophytou et al., 2021). For example, a comprehensive pan-cancer analysis demonstrated a strong association between the upregulation of immunosuppressive molecules like *PD-L1* and *CTLA-4*, and the downregulation of *PER1, PER2*, and *PER3* genes, underscoring the impact of disrupted clock genes on T cell exhaustion and immune evasion in the TME (Wu et al., 2019). In ovarian cancer, B lymphocyte, macrophage, and neutrophil infiltration levels were inversely correlated with *PER1* gene expression (Chen et al., 2021). Recently, a colon cancer research indicated that the epigenetic regulator *CBX4* was negatively associated with myeloid-derived suppressor cells and cancer-associated fibroblasts, and showed coordinated expression with *PER1* and *PER3* genes (Wei et al., 2021). Yang et al. (Yang et al., 2019) found a specific circadian rhythm in *PD-L1* expression and a positive correlation between the *PER1* gene and CD4^+^ and CD8^+^ T cell infiltration on lung cancer. Furthermore, in endometrial cancer, the *PER1* gene is linked to immunological factors like PD-1/PD-L1 and inflammatory markers such as TNF-α and IL-6 (Wang et al., 2020c). Additionally, chronic shift-lag-induced suppression of *Per1* and *Per2* genes impaired natural killer (NK) cell-mediated immunosurveillance and promoted tumorigenesis in mice, potentially due to decreased expression of immune functional receptors like Ly49D, Ly49G2, and Ly49H (Zeng et al., 2020). These studies robustly support the *PER* gene family’s regulatory role in immune cell function and tumor immune cell invasion.

## 8 The *PER* gene family and cancer therapy

To enhance patient prognosis, chemotherapy employs cytotoxic chemicals to eradicate cancer cells (van Stein et al., 2021). It may also serve as an adjuvant therapy alongside radiotherapy or surgery (Liu et al., 2022c). However, factors such as intratumor heterogeneity, adaptive mutations, epigenetic alterations, and metabolic changes enable some cancer cells to withstand clinical doses of medications, thereby escalating the challenge of medication resistance in cancer treatment (Vasan et al., 2019). Investigating the molecular mechanisms of drug resistance is crucial for improving therapeutic outcomes and introducing new treatment strategies (Nussinov et al., 2021; Liu et al., 2023b). Previous research revealed that oncogene-transformed mouse embryonic fibroblasts developed increased resistance to chemotherapeutic agents like methotrexate, gemcitabine, and etoposide, due to alterations of *Per2* gene. Wang et al. (Wang et al., 2020d) found a link between the downregulation of *PER2* gene expression in cisplatin-resistant ovarian cancer cells and the PI3K/AKT signaling pathway, which led to the activation of the multidrug resistance gene 1 (*MDR1*). Conversely, upregulating the *PER2* gene in cervical cancer cells inhibited the PI3K/AKT pathway, diminishing multidrug resistance protein production and enhancing cisplatin’s lethal effect on cancer cells (Wang et al., 2022). Additionally, Cai et al. (Cai et al., 2018) noted a downregulation of *PER3* gene in prostate cancer patients with paclitaxel-resistant, while the paclitaxel sensitivity of cancer cells could be rescued by overexpressing the *PER3* gene. Moreover, Redondo and colleagues observed that lower *PER2* gene expression increased the susceptibility of esophageal cancer cells to cisplatin, promoting higher rates of cell apoptosis (Redondo et al., 2021).

Radiation therapy is a prevalent cancer treatment modality. Increasing research focuses on the link between circadian rhythm and radiotherapy, given that specific circadian clock genes are vital for DNA repair and apoptosis induced by ionizing radiation and influence cell sensitivity to radiation at different cell cycle stages (Amiama-Roig et al., 2022). In glioma research, Zhu et al. (Zhu et al., 2019) observed that the downregulation of the *PER1* gene in U343 cells diminished X-ray-induced DNA damage and cell death through the CHK2-TP53 pathway. A comprehensive cohort study of 1,690 breast cancer patients demonstrated a significant association between the genotype variations of *PER3* gene and radiotherapy side-effects, suggesting the potential of the *PER3* gene as a predictor for radiotherapy response (Webb et al., 2022). Furthermore, studies have explored the impact of radiation timing on long-term prognosis and adverse patient outcomes, along with the predictive capacity of clock gene profiles for radiotherapy efficacy (Jin et al., 2021; Sapienza et al., 2021; Kong et al., 2022; Tang et al., 2023).

## 9 The *PER* gene family-related drugs

Several studies have focused on the *PER* gene family in cell and animal models to augment cancer therapy. For instance, Yang et al. (Yang and Stockwell, 2008) demonstrated that IC261, a CKⅠε inhibitor, impedes fibrosarcoma cell proliferation by stabilizing PER2 protein. Oshima et al. (Oshima et al., 2019) conducted a proteomic analysis and identified that the CKⅡ inhibitor GO289 inhibited the *PER2* phosphorylation sites, leading to extended circadian rhythms and suppressed growth in kidney cancer and acute myeloid leukemia cells. LY2857785, a CDK9 inhibitor, has been shown to decrease core clock protein levels including BMAL1 and PER2, by upregulating REV-ERBα expression (Ou et al., 2019). Additionally, mutations of PER2 could shorten circadian rhythms in mice induced by N-ethyl-N-nitrosourea (ENU) in the PAS domain, suggesting the PAS domain as target within the *PER* gene family for future interventions (Militi et al., 2016). These findings underscore the potential of targeting the *PER* gene family in cancer therapy. However, more clinical researches are necessary to elucidate their precise pharmacological effects and minimize unintended adverse impacts on healthy tissues.

## 10 Perspective and conclusion

**FIGURE 4:**
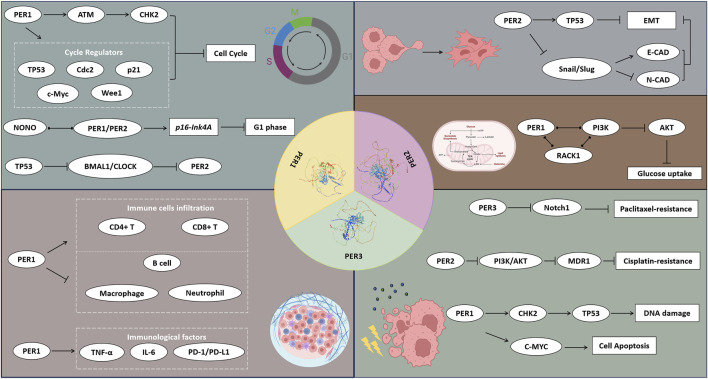
The potential mechanisms of PER1, PER2, and PER3 proteins affecting the hall markers of cancers. The PER1, PER2, and PER3 proteins influence the cell function, metabolism, TME, and therapy responses of cancers via a wide range of signal pathways and regulatory factors.

As a crucial component of clock genes, the *PER* gene family in human (*PER1*, *PER2*, and *PER3*) plays a pivotal role in numerous significant pathological processes, including cancer initiation and development. These genes, by modulating downstream gene expression, are instrumental in regulating cell cycle and invasion processes, altering tumor cell metabolism, impacting the tumor immune environment, and influencing treatment responses (Figure 4; Table 1). This modulation leads to varied clinical and pathological features and prognostic outcomes in cancer patients. The dysregulated proliferative phenotype is a universally recognized hallmark of cancer. Consequently, research on the *TP53* gene in cancer maintains a prominent and steadfast position, given its direct association with the cell cycle and proliferative phenotype, which may be intricately linked to circadian rhythms and the biological clock. Yet, the extent of the *PER* gene family’s influence on cancer cell cycles remains incompletely elucidated. In our review, we thoroughly reviewed the discovery history, fundamental biological functions, and the interplay between the *PER* gene family and various tumor characteristics. We also anticipate further studies on the relationship between the *PER* gene family and *TP53* to elucidate their connections. Our review advocates for in-depth investigations into the *PER* gene family’s role in specific cancer types or stages, and their molecular mechanisms, to identify potential biomarkers for cancer risk and prognosis.

**TABLE 1:** Main studies of associations between the *PER* gene family and cancers

Author	Year	Gene	Cancer type	Affect	Method	Function	Mechanism
Han et al. (2016)	2016	*PER1*	CHOL	Cell function	Overexpression of *PER1* in Mz-ChA-1 cells	Decreased cell proliferation, lower G2/M arrest, and enhanced cell apoptosis; Inhibition of tumor growth *in vivo*	miR-34a directly targets PER1
Wang et al. (2020b)	2020	*PER2*	CML	Cell function	Overexpression of *PER2* in KCL22 cells	Cell cycle arrest; inhibition of cell proliferation *in vivo* and *in vitro*	Not report
Guo et al. (2020)	2020	*PER2*	OSCC	Cell function	Overexpression of *PER2* in OSCC cells	Reduced cell migration and invasion; Suppresses tumor metastasis	Activation of downstream signals of TP53 and EMT regulatory genes
Gao et al. (2021)	2021	*PER1 PER2 PER3*	GBMLGG	Cell function	Downregulation of *PER1*, *2*, 3 after IDH1 mutation in U87-MG cells	Decreased S phase-associated proteins; Increased G1 phase-associated proteins	Activation of Smad pathway
Xiong et al. (2022)	2022	*PER2*	COADREAD	Cell function	Downregulation of *PER2* in RKO cells	Cell migration promotion	Activation of the Snail/Slug axis through inhibiting TP53
Papagiannakopoulos et al. (2016)	2016	*PER2*	LUAD	Metabolism	Genetically engineered mouse model of lung adenocarcinoma with *PER2* knockout	Increased glycolysis and utilization of glucose	Not report
Gong et al. (2021)	2021	*PER1*	OSCC	Metabolism	Overexpression of *PER1* in SCC15 cells	Inhibited glycolysis and cell proliferation	Inhibition of PI3K/AKT pathway
Yang et al. (2019)	2019	*PER1*	LUAD, LUSC	Tumor immunity	Computational estimation based on TCGA data	Positive correlation with CD4 T cells	Not report
Wang et al. (2020c)	2020	*PER1*	UCEC	Tumor immunity	Overexpressing *PER1* in EC ishikawa cells	Increased expression of TNF-a, IL-6, and PD-1/PD-L1; Promotion of apoptosis; Inhibition of tumor invasion	Inhibition of TNF-α/TNFRSF6B pathway
Chen et al. (2021)	2021	*PER1*	OV	Tumor immunity	TIMER database and CIBERSORT algorithm	Positive correlation with neutrophils, regulatory T cells, and M2 macrophages	Not report
Cai et al. (2018)	2018	*PER3*	PRCA	Chemotherapy	Overexpression of *PER3* in prostate cancer-resistant cell lines	Reduced IC50 to paclitaxel; Cell cycle arrest; ncreased cell apoptosis	Inhibiting the Notch pathway
Wang et al. (2020d)	2020	*PER2*	OV	Chemotherapy	Decrease in *PER2* expression in SKOV3/DDP cells via methylation of CpG promoters	Higher expression of multidrug resistance-related protein 1 (MRP1) in SKOV3/DDP cells-derived xenografts in mice	Inhibiting the PI3K/Akt signaling pathway and drug-resistance factors
Redondo et al. (2021)	2021	*PER2*	ESCA	Chemotherapy	Downregulated of *PER2* after dexamethasone synchronization in KYSE-410 cells	More cisplatin induced-DNA damage; Higher cell apoptosis	Not report
Wang et al. (2022)	2022	*PER2*	CESC	Chemotherapy	Overexpression of *PER2* in Hela/DDP and SiHa/DDP cells	Inhibition of tumor growth and proliferation in mice treated with cisplatin; Increased cell apoptosis	suppressing PI3K/AKT pathway
Zhu et al. (2019)	2019	*PER1*	GBMLGG	Radiotherapy	Downregulation of *PER1* in U343 cells	Reduced DNA damage after X-ray irradiation; Lower cell death rate	Inhibiting the CHK2-TP53 signaling and proapoptotic processes

In future studies, our focus on the *PER* gene family will encompass several key areas (Feng et al., 2023a): Developing personalized treatment strategies: By harnessing a thorough comprehension of the *PER* genes’ expression and functional disparities across different cancer types, we aim to craft more targeted treatment methodologies to improve patient responses and survival rates (Hanahan, 2022); Integrating immunotherapy: This involves exploring how immunotherapy can enhance the immune system’s ability to detect and eliminate tumors, potentially including the creation of immunotherapeutic approaches directly linked to *PER* genes regulation (Shen et al., 2022); Deciphering treatment resistance mechanisms: Conducting detailed analyses of the resistance mechanisms associated with the *PER* genes to ascertain why some patients resist certain treatments, thereby providing insights for novel therapeutic development (Wang et al., 2023); Discovering early diagnostic and predictive biomarkers: Leveraging insights into the regulatory roles of *PER* genes in various biological processes to identify novel early diagnostic or predictive biomarkers, aiming to improve patient outcomes (Feng et al., 2023b); Fostering multidisciplinary collaboration and technological innovation: By enhancing collaboration across disciplines and integrating cutting-edge technologies from bioinformatics, molecular biology, and immunology, we plan to conduct an in-depth and comprehensive exploration of the mechanisms of action of *PER* genes in cancer.
